# Can Celiac Disease Be Prevented?

**DOI:** 10.3389/fimmu.2021.672148

**Published:** 2021-05-14

**Authors:** Renata Auricchio, Riccardo Troncone

**Affiliations:** Department of Medical Translational Sciences & European Laboratory for the Investigation of Food-Induced Diseases, University Federico II, Naples, Italy

**Keywords:** risk factors, biomarkers, celiac disease, children, gluten

## Abstract

Celiac disease (CD) is an autoimmune disorder triggered by gluten in genetically susceptible individuals characterized by a variable combination of gluten-dependent symptoms, presence of specific autoantibodies and enteropathy. The health burden of CD is considerable, as it reduces quality of life  and, at a societal level, has extensive negative economic consequences. Prevention strategies are based on the identification of at-risk subjects and identification and elimination of risk factors. A number of prospective observational and interventional studies conducted on the general population, and more often in subjects at-risk, have given important information on the natural history of the disease. Both genetic and environmental factors have been identified with the former, in particular histocompatibility genes, playing a major role. Environmental factors, some operating already before birth, have been identified, with feeding pattern in the first year of life (breast feeding, amount and time of introduction of gluten) and infections being the most relevant. Prospective studies have also allowed the identification of biomarkers predictive of the disease which in perspective could better define the population on which to intervene. Interventions have been so far limited to modifications of feeding patterns. However, as also learnt from diseases that share with CD genetic risk factors and mechanisms of damage, such as type 1 diabetes (T1D), future strategies may be envisaged based on protection from infections, manipulation of microbiota, intervention on T cells.

## Introduction

Celiac disease (CD) is an immune mediated systemic disorder elicited by gluten and related prolamines in genetically susceptible individuals and characterized by a variable combination of gluten-dependent clinical manifestations, anti-tissue transglutaminase antibodies positivity and enteropathy (1). The disease results from a complex interplay between genetic, environmental and immune factors leading to an inappropriate mucosal T cell response to gluten and eventually to a remodeling of the small intestinal mucosa (villous atrophy) and its clinical consequences (2).

CD is a frequent disorder. A recent meta-analysis has indicated the pooled global prevalence of CD being 1.4%, based on positive results from tests for anti–tissue transglutaminase and/or anti-endomysial antibodies (called seroprevalence) and 0.7% that of biopsy-confirmed CD (3). The prevalence goes up to 10% in at risk groups, such as children and adolescents with first-degree relatives with CD, patients with autoimmune diseases (e.g. type 1 diabetes, T1D), IgA deficiency and chromosomopathies. It is also worthy to note that the incidence has increased over the past several decades emphasizing the relevance of environmental factors (4).

The health burden of CD is considerable, as it reduces quality of life  (5, 6) and in social life has extensive negative consequences. The costs of a particular disease in different Countries depends on the structure of a particular healthcare system. However, from all Countries there are reports of significant additional primary care costs associated with CD. When compared with other chronic illnesses, the costs of patients with CD were similar to those of patients with diabetes and hypertension (7). For these reasons primary prevention has become a priority in the research agenda of CD.

**Figure 1:**
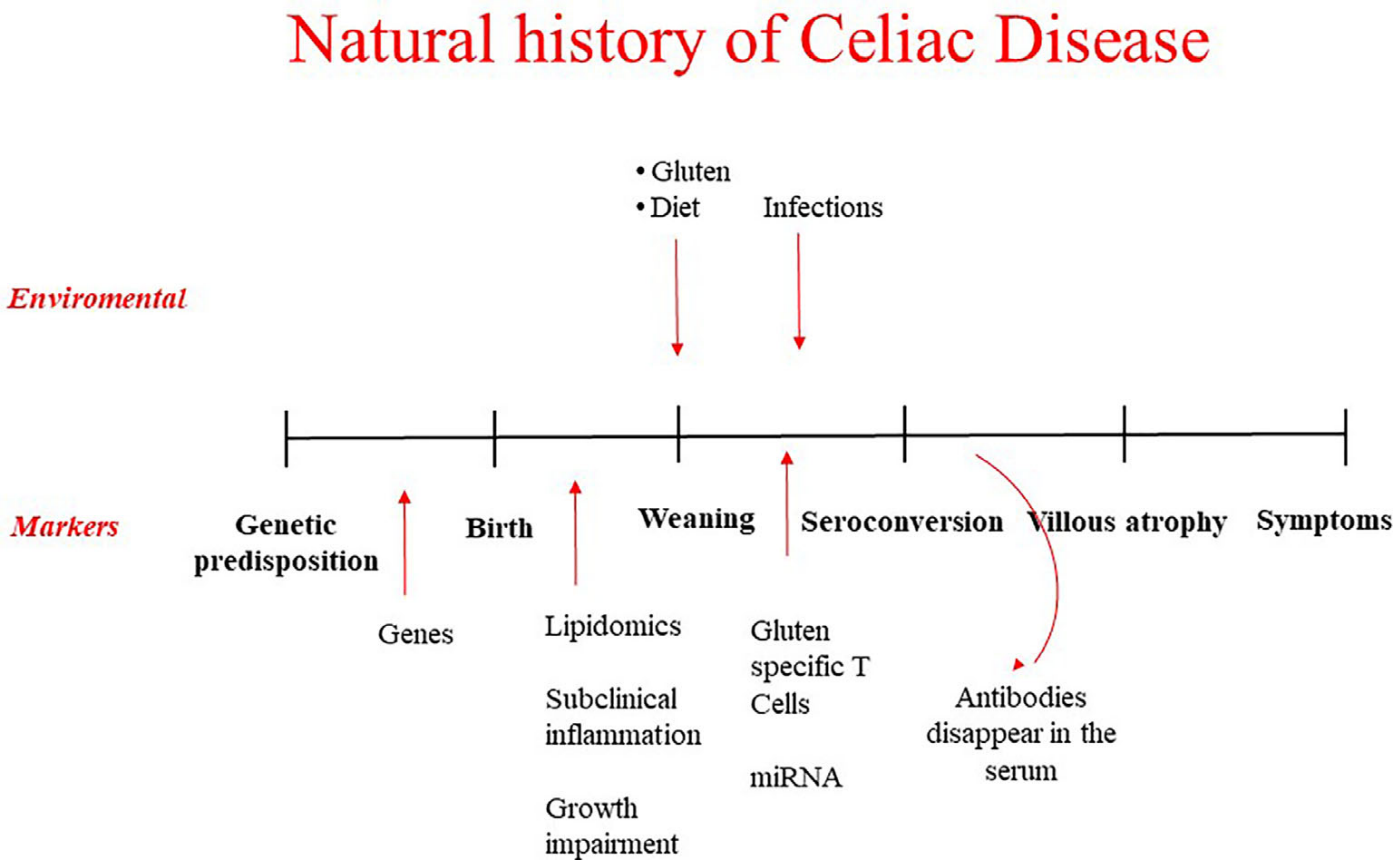
Natural history of celiac disease: environmental factors possible target of prevention strategies and biomarkers to identify at risk children.

A strategy for primary prevention is based on the identification of at risk subjects primary target of the intervention and on the identification of environmental factors that favor disease development whose manipulation may decrease the risk. Recently a number of prospective studies have shed light on these aspects, in a first place helping to understand the natural history of the disease. Different phases have been identified, from the genetic predisposition with HLA alleles contributing to 30-50% of the genetic risk, to the pre-weaning phase where environmental factors may play a role even before birth, to the introduction of gluten in the diet, to the development of CD-specific autoantibodies, to the eventual development of small intestinal mucosal damage and the consequent clinical manifestations (**Figure 1**). At each of this stage it is possible to intervene to prevent the gluten-induced harm. Early diagnosis through screening policies based on the detection of CD-associated autoantibodies and efforts to assure compliance with the gluten free diet represent the basis for secondary and tertiary prevention of CD. However, this mini-review will mainly focus on primary prevention and on the possible strategies to halt the disease process before mucosal damage occurs.

### The Target: Biomarkers to Identify at Risk Subjects

Those subjects who are at the highest risk to develop CD are the first target of intervention strategies. Much effort has been made in recent years trying to identify possible biomarkers that would allow clinicians to classify individuals based on their risk of developing CD before any clinical and serological signs of the disease.

More than ninety percent of CD patients have the HLA-DQ2.5 haplotypes (encoded by the DQA1*0501 and DQB1*0201 alleles) either in cis or trans position, the remaining showing the HLA-DQ8, DQ2.2 or DQ7 haplotypes (8). Furthermore, it is known that the risk among genetically predisposed first degree relatives increases up to 20% (9). However, not all DQ2 or DQ8 positive patients have the same probability to develop the disease, a gene dose effect due to the number of copies of DQB1*0201 having been reported (10). Studies in cohorts of children at risk for CD have prospectively confirmed these observations. In the European multicentre prospective study PREVENT-CD it was in fact shown that subjects homozygous for HLA DQ2 were those with the highest incidence of CD (11). Similar data were also produced by the Italian multicentre study, known as CELIPREV (12), and in the TEDDY study (13). Girls seem to be at higher risk to develop the disease. The contribution of non-HLA genes to the risk is less defined, but scores that take into account the contribution of single nucleotide polymorphisms related to CD have been developed (14) and could help in a more precise assessment of the genetic risk.

Prospective studies have helped to identify biomarkers predicting the development of the disease and then helping to identify subjects candidate to prevention strategies. Lipidomic modifications were considered as an early predictive marker for CD. In fact, it has been shown that since a very early age, even before the introduction of gluten, genetically predisposed children who will develop the disease during their life show a specific profile of phospholipids. A limited number of alkylacyl-phosphatidylcholine and lyso-phosphatidylcholine, together with the duration of breastfeeding, allows the discrimination of infants who will develop CD from those at a similar genetic risk who will not develop the disease (15). Interestingly similar observations have been made in T1D (16). These changes seem to be present already at 4 months of age, suggesting they are the result of very early phenomena, even in the gestational period, which may predispose to development of the disease in at risk subjects. In another study conducted in the DIPP cohort increased amounts of triglycerides of low carbon number and double bond count and decreased levels of phosphatydilcholine were noted before gluten introduction in subjects who later developed CD; in this case the changes were attributed to impaired lipid absorption (17).

Before seroconversion other features are reported in infants who will later develop the disease. In fact, Galatola et al. have shown that a small set of non-HLA genes is differently expressed in subjects at risk who then develop the disease already in the first year of life, long before the appearance of other clinical and serological signs of the disease (18).

Also microRNAs have been considered as possible biomarkers capable of predicting disease. In fact, some studies have shown that both at the tissue and blood level there are microRNA profiles able to distinguish celiac patients from controls (19).

The seroconversion i.e. the appearance in serum of anti-tissue transglutaminase antibodies and other autoantibodies related to CD is a major step of the natural history of the disease. However, they are not invariably associated to the mucosal damage; moreover, they do not automatically lead to evolution towards villous atrophy. On the contrary in percentages ranging from 30 to 90% antibodies can disappear from the serum (20, 21) indicating this condition of potential CD (normal mucosa but presence of autoantibodies) still represent a situation amenable to prevention, being possible to halt the progression to the full blown disease.

### Identification of Risk Factors and Intervention on at Risk Infants: Feeding in the First Years of Life

Breastfeeding has for long time been considered the main protective factor for the development of CD (22). More recently, the evidence coming from most studies (23) including the two large interventional studies on children at risk for CD (11, 12), have concluded that exclusive or any breastfeeding, as well as breastfeeding at the time of gluten introduction, did not reduce the risk of developing celiac disease during childhood.

In recent past, studies had identified a “window of tolerance”: gluten introduction between 4 and 6 months of life, was hypothesized to reduce the risk of developing CD (24). More recent studies on prospective cohorts at risk for CD such as PREVENT CD (11), CELIPREV (12) have instead shown that there is no substantial difference in the incidence of the disease whether gluten is introduced early (4 months) or later (after the 12th month of life). Other observational studies both in general population and in at risk groups for T1D also reached the same conclusions. Therefore, the current guidelines of the European Society for Gastroenterology, Hepatology and Nutrition (ESPGHAN) recommend introducing gradually gluten between 4-12 months of life (25). Recently, several studies have been published describing the effect of challenging young babies with allergen containing foods at a very early age to induce lifelong tolerance. In this context data coming from the EAT study, an open labelled randomized clinical trial aimed to assess if early introduction of six allergenic foods can prevent food allergy (26), showed that introduction of gluten at 4 months was associated with reduction of CD prevalence. The small size and the criteria used for diagnosis suggest caution in the interpretation of these results.

Whether the amount of gluten at the time of weaning and in the first year of life can in any way influence the risk of CD has been the focus of different studies. In the PREVENT CD study it was shown that gluten consumption pattern, as well as the amount of gluten consumed in the first three years of life, do not influence the development of CD (27), although a trend to a positive effect was noted in the subgroup at lower genetic risk, suggesting that effect related to the gluten amount may become more visible in those with lower genetic risk. In fact, more recent data have been published on two cohorts at risk for T1D [TEDDY (28), DAISY (29)] and one on the Norwegian mother-child cohort (30), suggesting that gluten ingested (around 3-10 g/day) is associated with the development of autoimmunity to T1D and CD. Taken together all these studies, it could be concluded that one extra slice of bread (2 g/day of gluten) could cause 20-50% increase in the risk of CD. Interestingly, it is likely that the amount of gluten may concur with other risk factors (in particular infections in the first years of life) to the development of CD. This is suggested by a recent observation in the context of the TEDDY study of an additive effect of more than 10 g/day of gluten ingested and virus infection (31).

The idea that gluten is not the only nutritional factor for the development of the disease is gaining ground in the recent years, but rather it could be that specific dietary pattern could cause a basal inflammatory state increasing the susceptibility to chronic diseases, such as CD. Roager et al. have shown that a diet rich in whole grain is able to reduce the inflammatory state of adult obese patients (32). Likewise, the work of Barroso et al. showed that a “prudent” diet at one year of life, with more fruit, vegetables, vegetable oil and cereals and less snacks, confectionary and sugars, in other words more like the Mediterranean diet, is able to reduce celiac autoimmunity at 6 years (33).

### Identification of Risk Factors and Intervention on at Risk Infants: Infections, Vaccines, Manipulation of Microbiota

Another important hypothesis is that the process of autoimmunity leading to CD is stimulated or switched on by the occurrence of common infections during the period preceding its onset. The authors of several studies have suggested that early infections might contribute to the risk of developing CD. First of all, repeated infections by Rotavirus (34) and *Parechovirus* (35) were associated to the risk of CD. More recently, *Reovirus* infection was also indicated in association to CD (36). In the TEDDY study, gastrointestinal infections increased the risk of celiac autoimmunity by 33% in genetically predisposed children in the following 3 months of life (37). In the Norwegian Mother and Child Cohort Study, children with more than 10 infections before 18 months of age had a significantly higher risk of developing CD later in life than children with less than four infections (38). Subsequently, in the same cohort it was shown that the increased risk for CD was associated with gastrointestinal infection mainly caused by *Enteroviruses*, especially if infection was contracted before seroconversion (39). The increased risk associated with *Enterovirus* was recently confirmed in TEDDY study by Lindorfs et al. (31). Moreover, they hypothesized that there is a cumulative effect of enteroviral exposure and higher amount of gluten consumed in the first 2 years of life (31). Interestingly, also non-gastrointestinal infections were associated to the risk of CD in an Italian prospective cohort study: a higher frequency of respiratory tract infections among CD patients during the first 24 months of life significantly contributed to discrimination of case *versus* controls (40).

Given the important role of infections, vaccination has been indicated as a strategy for prevention. In fact, several studies have shown a reduced incidence of disease in subjects vaccinated for *Rotavirus* (37, 41). Furthermore, a trial is being conducted with the use of an anti-*Coxsackie* vaccination for the prevention of T1D and CD (Clinical Trial n° NCT04690426).

A number of studies have addressed the potential role of microbial composition in the evolution of CD. High risk children have been shown to possess a different microbiota in comparison to children with no or low genetic risk for CD (42). Contrasting data have been produced on the existence of an early microbial signature in infants from at risk groups who later progress to CD (43, 44). Manipulation of the microbiota has then become another possible strategy. Probiotics are candidate for their proven anti-inflammatory effects; but controversial results have been published about their ability to prevent CD (45). One ongoing clinical trial (NCT3562221) in this context addresses the effects of GFD and probiotics during the first 3 years of life on the development of celiac disease. It is clear that, also because the low risk of side effects, that will be one of the area of major development.

Finally, the identification of other risk factors could also be important in the design of prevention strategies based on multiple interventions. However, given also the relatively little impact of each of them, conflicting results have been so far obtained for example in relation to the use of antibiotics and the modalities of delivery (46).

### Halting the Progression of CD: Analogies With Type 1 Diabetes

CD is now considered by most an autoimmune disease. It is often associated with other autoimmune diseases, first among all T1D. With T1D, CD shares genes conferring risk (both HLA and non HLA) (47) and immunological mechanisms inducing damage at the target tissue (intestinal mucosa or pancreatic islets). Lessons from what implemented for T1D prevention may be particularly useful for CD. Also for T1D the target population for primary prevention trials is individuals who carry high risk genotypes before the first appearance of islet autoantibodies and also for T1D these trials include mostly low risk dietary interventions such as the avoidance of cow’s milk or gluten and the supplementation of n-3 fatty acids or vitamin D (47). The results of these trials have been so far quite disappointing. None of these specific dietary factors has been proved to be a definite risk factor inducing beta cell autoimmunity. More recently primary prevention has started focusing on the modulation of the immune system by antigen specific immunotherapy, such as oral insulin (48). Furthermore, the observation that as in CD certain viral infections e.g., *Coxsackie B* may promote autoimmune attack to pancreatic islets has prompted efforts to develop vaccines that are going to be tested in clinical trials.

Most of the efforts in T1D have been so far directed to slow or to halt the progressive beta cell destruction. Nicotinamide, antigen-specific immune therapy (oral and nasal insulin, GAD alum), monoclonal antibodies, immunosuppressive drugs, hydroycloroquine and anti-inflammatory agents have been tested (47). Particularly promising is the use of monoclonal antibody to CD3 (Tepluzimab) targeting CD8+ cells responsible of beta cell destruction in T1D (49). Here the analogies with potential CD (anti tissue transglutaminase antibodies, anti-TG2) present in the absence of mucosal damage) are very strong. Moreover, we know from clinical studies that the appearance of anti-TG2 antibodies in CD is still reversible, as shown by observations in infants born in celiac families and subjects with T1D (see above). That suggests that also in the presence of anti-TG2 antibodies the evolution to villous atrophy is not obligatory; in theory it may still be prevented as the experience with anti-CD3 in pre-diabetes seems to indicate (49). Other possibilities are based on the induction of specific tolerance to gliadin through “vaccination” with gliadin peptides (50) or use of nanoparticles containing gliadin peptides (51). Another attempt has been based on the use of probiotics. A randomized double blind placebo-controlled trial of *Lactobacillus plantarum* and *Lactobacillus paracasei* to suppress celiac autoimmunity in infants at risk has led to a decrease of anti-TG2 antibodies titres and to changes of the phenotype of PBMC (52). In general, one of the challenges of such trials is to balance the potential benefits against the risks, and this is particularly true when it comes to strategies which alter the immune response.

Another important indication that seems to come from studies on T1D is that further attempts should not concentrate on single hypothesis or intervention. It is very likely that a combination of different factors may be decisive, as suggested by the additive risk given by the amount of gluten and viral infections (31), and therefore a combination of different approaches could be the next strategy.

## Conclusions

The pathogenesis of CD need to be clarified: environmental factors and genetic factors need to be better understood. Prospective studies have much improved our knowledge of the natural history and have provided biomarkers that help to define the different level to which intervene. Primary prevention remains the main goal to achieve, with interventions planned as early as possible even before birth. On the other hand, even if CD associated autoantibodies develop there is still potential therapeutic benefit from intervention to halt and possibly reverse the disease. We acknowledge that in the case of CD prevention is moving its first steps. Attempts based on the timing of gluten introduction in infants’ diet are inconclusive. We are now waiting for the results of trials based on antiviral vaccinations. However, there is no doubt that studies aimed to find a prevention strategy for CD will represent in the next years one of the frontiers of the research in CD with important consequences also in the management of other autoimmune diseases.

## Author Contributions

RA and RT both contribute to select papers and write the review. All authors contributed to the article and approved the submitted version.

## Conflict of Interest

The authors declare that the research was conducted in the absence of any commercial or financial relationships that could be construed as a potential conflict of interest.
